# Association of triglyceride-glucose index levels with gestational diabetes mellitus in the US pregnant women: a cross-sectional study

**DOI:** 10.3389/fendo.2023.1241372

**Published:** 2023-10-10

**Authors:** Yan Zeng, Li Yin, Xiaoping Yin, Danqing Zhao

**Affiliations:** ^1^ Department of Obstetrics and Gynecology, Affiliated Hospital of Guizhou Medical University, Guiyang, China; ^2^ Guiyang Maternal and Child Health Care Hospital, Guiyang Children’s Hospital, Guizhou Medical University, Guiyang, China

**Keywords:** triglyceride-glucose index, gestational diabetes mellitus, cross-sectinal study, NHANES, insulin resistance

## Abstract

**Objective:**

This investigation aimed to assess the correlation between the triglyceride-glucose (TyG) index and gestational diabetes mellitus (GDM) in pregnant women in the United States.

**Methods:**

We calculated the TyG index utilizing data from pregnant women who participated in the National Health and Nutrition Examination Survey (NHANES) through 1999 to March 2020, and then employed multivariate logistic regression, smoothed curve fitting, and subgroup analysis to investigate the association between the TyG index and gestational diabetes during pregnancy.

**Results:**

Logistic regression models revealed a positive association between the TyG index and GDM, remaining significant even after adjusting for all confounding variables (OR=3.43, 95% CI: 1.20-9.85, *P* = 0.0216). Subgroup analysis demonstrated consistent correlations and showed that there is no difference in the TyG index among first trimester subgroup. The TyG index had limited diagnostic efficacy for GDM (AUC=0.57, 95% CI: 0.50-0.63).

**Conclusion:**

The TyG index correlates positively with the GDM, however its diagnostic efficacy is limited. Further research on the TyG index as an early predictor of GDM is required.

## Introduction

Gestational diabetes mellitus (GDM) refers to varying degrees of glucose intolerance that occur or are identified for the first time during pregnancy, irrespective of pre-existing diabetes (1). Over the last few years, the incidence of GDM has gradually increased, ranging from 9.3% to 25.5% (2). GDM is often associated with preeclampsia, macrosomia, perinatal anomalies, and mortality, while being closely linked to the onset of metabolic syndrome and hyperglycemia in both mother and offspring (3). This condition significantly affects the well-being of pregnant women and fetuses and poses a concealed risk for future ailments (4, 5). Clinical diagnosis of GDM typically occurs during the 24-28 week gestational period using a 75g oral glucose tolerance test (OGTT) (6). However, empirical evidence suggests that by the time GDM is diagnosed at this stage, both the mother and fetus may have already been adversely affected to varying degrees, despite the potential benefits of symptom management (5, 7). Thus, early recognition of pregnancies at risk for GDM is essential in preventing negative outcomes for pregnancy and intergenerational transmission of metabolic disorders.

Early detection of insulin resistance (IR) in pregnant women has been shown to assist in predicting the onset of GDM before clinical diagnosis (8, 9). The TyG index, calculated from fasting plasma glucose (FPG) and serum triglycerides (TG), is considered a straightforward, economical, replicable, and reliable surrogate for IR (10, 11). While many studies have investigated the relationship between the TyG index and GDM, suggesting its potential as an early GDM risk indicator (12, 13), there may be variations between ethnic groups. For instance, Sánchez-García et al. (14) found no significant difference in TyG index values between Latin American pregnant women with and without gestational diabetes. A meta-analysis by Song et al. (15) indicated that a higher TyG index may predict GDM in Asian women but not in non-Asian women. Therefore, using data from the National Health and Nutrition Examination Survey (NHANES), we conducted a cross-sectional investigation with a population of pregnant women in the United States to assess the connection between the TyG index and GDM.

## Materials and methods

### Study populations

The present investigation utilized the NHANES database, covering data from 1999 through March 2020. NHANES was originally designed to collect comprehensive data on the nutritional and health conditions of adults and children in the United States, employing a complex multi-stage random sampling process for its surveys. The study protocols for these surveys were authorized by the National Center for Health Statistics Ethics Review Committee, and all participants provided informed consent before data collection. For more detailed information, refer to http://www.cdc.gov/nchs/nhanes/index.htm.

The study’s cohort comprised women between the ages of 20 and 44 years (n=1469) who had positive urine test result for human chorionic gonadotropin (hCG). Participants lacking FPG and TG data (n=781), as well as those diagnosed with diabetes or using diabetic medication or insulin (n=27), were excluded. Ultimately, the final sample size consisted of 661 individuals.

### Measurements and definitions

Samples of blood were collected in the morning hours after an 8.5-hour fast and processed to determine the concentrations of fasting blood glucose and fasting total triglycerides with an automatic biochemical analyzer. The TyG index was calculated using the formula: Ln [TG (mg/dL) × FPG (mg/dL)/2] (11). GDM was ascertained according to the fasting plasma glucose threshold of 5.1 mmol/L from the strategy recommended by the International Association of Diabetes and Pregnancy Study Groups (IADPSG) Consensus Panel (16) and the American Diabetes Association’s one-step OGTT (17) for the identification and evaluation of hyperglycemia conditions in pregnancy.

### Covariates

This study incorporated various covariates that could potentially influence the association between the TyG index and the risk of developing GDM. The demographic variables considered were age, race, education level, poverty income ratio (PIR), body mass index (BMI), hypertension, hypercholesterolemia, smoking status, alcohol consumption, total cholesterol (TC), high-density lipoprotein cholesterol (HDL-C), low-density lipoprotein cholesterol (LDL-C), glycohemoglobin (HbA1c), and self-reported gestational age.

### Statistical analysis

Continuous variable data was presented as mean ± SD (standard deviations), while categorical variables were represented as percentages. To assess differences in baseline characteristics between the non-GDM and GDM groups, the Kruskal-Wallis H test (for continuous variables) and the chi-square test (for categorical variables) were used. The logistic regression model was then applied to evaluate the association between the TyG index and GDM. Multiple models were used to measure the odds ratio (OR) and 95% confidence interval (CI) while adjusting for confounding factors. The first model (crude model) did not include any covariate adjustments, while Model 1 accounted for age and race. Model 2 included additional adjustments for education level, BMI, PIR, HDL-C, LDL-C, TC, HbA1c, gestational age, hypertension history, and hypercholesterolemia history, building upon the adjustments made in Model 1. Furthermore, the TyG index was divided into tertiles, with the first tertile serving as the reference for trend analysis. Three models were employed for multivariate analyses, controlling for variables and fitting a smooth curve. Subgroup analyses were conducted based on age, race, education level, BMI, hypertension status, hypercholesterolemia status, and gestational age using stratified multivariate regression analysis. Log-likelihood ratio analysis was performed to assess interaction and investigate heterogeneity of connections among subgroups. A *P* value < 0.05 was considered statistically significant. To determine the diagnostic effectiveness of the TyG index for GDM, the receiver operating characteristic (ROC) curve was used and the area under the ROC curve (AUC) was calculated to quantify its screening value. All statistical analyses were conducted using R packages 3.4.3 and EmpowerStats software 4.1.

## Results

### Baseline characteristics of the participants

The present study comprised 661 pregnant women with an average age of 28.01 ± 5.30 years. Among the participants, 119 (18%) were diagnosed with GDM. **Table 1** presents a comprehensive comparison between non-GDM and GDM pregnancies. The occurrence or absence of GDM showed significant associations with age, BMI, drinking status, gestational age, TC, LDL-C, HDL-C, HbA1c, and TyG index (*P* < 0.05). Compared to non-GDM pregnant women, those with GDM were characterized by advanced age, abstinence from alcohol consumption, lower levels of TC and LDL-C, higher HbA1c and BMI, and elevated levels of TyG index.

**Table 1:** Baseline characteristics of participants.

Characteristics	Non-GDM (n=542)	GDM (n=119)	*P*-value
Age (years)	27.73 ± 5.12	29.28 ± 5.93	0.014
Poverty income ratio (PIR)	8.51 ± 23.49	8.91 ± 24.35	0.940
Fasting Glucose (mg/uL)	80.85 ± 6.22	100.45 ± 9.47	< 0.001
Total Cholesterol (mg/dL)	225.44 ± 50.65	199.92 ± 52.82	< 0.001
HDL Cholesterol (mg/dL)	68.32 ± 16.49	61.62 ± 15.41	< 0.001
LDL cholesterol (mg/dL)	122.05 ± 39.98	102.97 ± 34.87	< 0.001
Triglyceride (mg/dL)	165.23 ± 80.60	161.82 ± 100.85	0.220
HbA1c (%)	4.93 ± 0.31	5.16 ± 0.36	< 0.001
Race, n (%)			0.179
Mexican American	152 (28.04%)	29 (24.37%)	
Other Hispanic	34 (6.27%)	8 (6.72%)	
Non-Hispanic White	245 (45.20%)	46 (38.66%)	
Non-Hispanic Black	72 (13.28%)	21 (17.65%)	
Other Race (including multi-racial)	39 (7.20%)	15 (12.61%)	
Education level, n (%)			0.628
< High school	125 (23.06%)	25 (21.01%)	
≥ High school	417 (76.94%)	94 (78.99%)	
Body Mass Index, n (%)			< 0.001
< 25 (kg/m^2^)	176(32.47%)	16 (13.45%)	
≥ 25 (kg/m^2^)	366 (67.53%)	103 (86.55%)	
Smoking status, n (%)			0.702
Now	46 (8.49%)	12 (10.08%)	
Former	120 (22.14%)	29 (24.37%)	
Never	376 (69.37%)	78 (65.55%)	
Drinking status, n (%)			< 0.001
Mild	332 (61.25%)	0 (0.00%)	
Moderate	58 (10.70%)	0 (0.00%)	
Heavy	26 (4.80%)	4 (3.36%)	
Never	89 (16.42%)	102 (85.71%)	
Unclear	37 (6.83%)	13 (10.92%)	
Hypertension, n (%)			0.147
Yes	38 (7.01%)	13 (10.92%)	
No	504 (92.99%)	106 (89.08%)	
Hypercholesterolemia, n (%)			0.021
Yes	192 (35.42%)	29 (24.37%)	
No	350 (64.58%)	90 (75.63%)	
Gestational age, n (%)			<0.001
1^st^ Trimester	82 (15.13%)	21 (17.65%)	
2^nd^ Trimester	175 (32.29%)	20 (16.81%)	
3^rd^ Trimester	169 (31.18%)	30 (25.21%)	
Unclear	116 (21.40%)	48 (40.34%)	
TyG index	8.68 ± 0.52	8.82 ± 0.65	0.023

### TyG index and GDM correlation in various models

The logistic regression models were used to examine the correlation between different TyG index levels and GDM. In the crude model (**Table 2**), a significant positive association was observed between the TyG index and GDM (OR=1.16, 95% CI: 1.11-2.35, *P*=0.0124). After full adjustment in Model 2, this positive association remained consistent (OR=3.43, 95% CI: 1.20-9.85, *P*=0.0216), indicating that each incremental unit of the TyG index was associated with a 2.43-fold increased risk of gestational diabetes.

**Table 2:** Logistic regression analysis for the relationship between various TyG index and GDM in different models.

Parameters	Crude Model OR (95%CI), *P*-value	Model 1 OR (95%CI), *P*-value	Model 2 OR (95%CI), *P*-value
TyG index	1.61 (1.11, 2.35) 0.0124	1.88 (1.25, 2.81) 0.0022	3.43 (1.20, 9.85) 0.0216
TyG index Tertile			
Tertile 1	1.0	1.0	1.0
Tertile 2	0.90 (0.54, 1.51) 0.6942	0.98 (0.57, 1.66) 0.9317	1.62 (0.69, 3.80) 0.2660
Tertile 3	1.49 (0.93, 2.41) 0.0983	1.75 (1.04, 2.93) 0.0353	3.92 (1.16, 13.25) 0.0282
*P* for trend	1.46 (0.92, 2.30) 0.1067	1.68 (1.02, 2.76) 0.0399	3.22 (1.07, 9.72) 0.0379

Crude model adjusts for: none.

Model 1 adjusts for: age and race.

Model 2 adjusts for: Model 1+ BMI; education level; hypertension; hypercholesterolemia; PIR; HDL-C; LDL-C; TC; HbA1c; gestational age; smoking status and drinking status.

For sensitivity analysis, we categorized the TyG index into tertiles. In Model 2, the OR for Tertile 3-TyG index was 3.92 (CI: 1.16-13.25, *P*=0.0282) compared to Tertile 1-TyG index, representing a significant 2.92-fold increase in the likelihood of GDM in Tertile 3. However, there was no statistically significant difference between Tertile 1 and Tertile 2 (OR=1.62; 95% CI: 0.69–3.08; *P*=0.2660).

**Figure 1:**
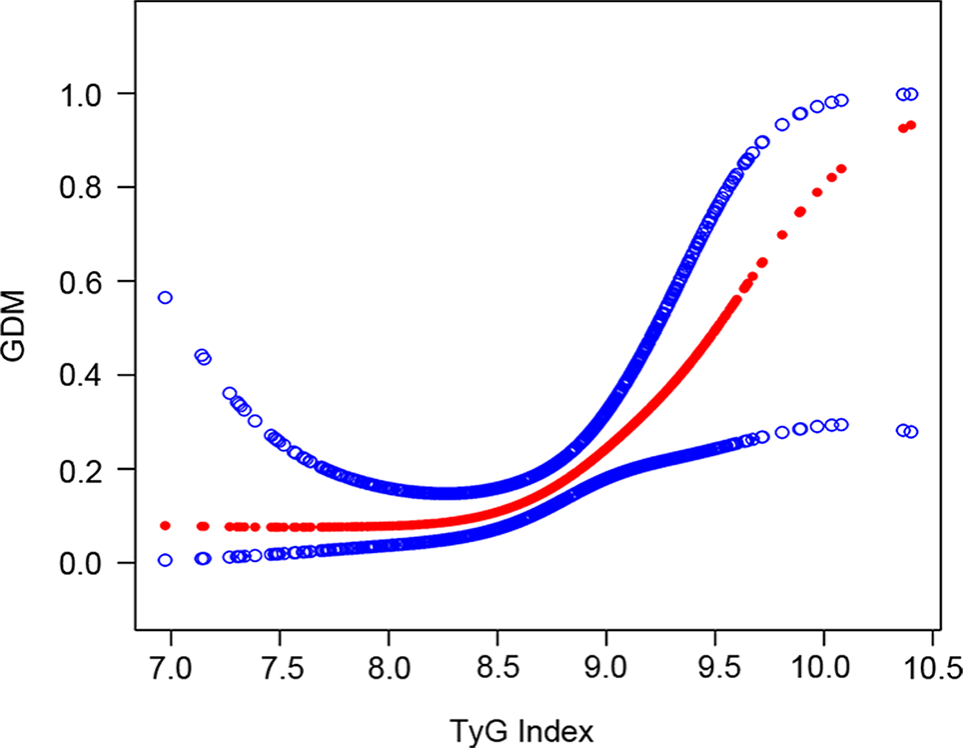
The association between TyG index and GDM. The solid red line represents the smooth curve fit between variables. Blue bands represent the 95% confidence interval from the fit.

The results of the smoothing curve fitting further demonstrated a positive association between the TyG index and the likelihood of GDM incidence, as shown in **Figure 1** (*P* for nonlinearity > 0.05).

### Subgroup analysis

The findings of our subgroup analysis indicate that the associations among the TyG index and GDM were inconsistent, as presented in **Table 3**. This was observed across subgroups stratified by age, race, education level, PIR, BMI, hypertension, hypercholesterolemia, and gestational age. However, interaction testing revealed that these parameters had no significant effect on the correlation that existed between gestational diabetes and the TyG index (all *P* values for interaction>0.05).

**Table 3:** Subgroup analysis of the associations between the TyG index and GDM.

Subgroups	OR (95%CI), P value	*P* for interaction
Age (years)		0.9023
<35	3.46 (1.16, 10.33) 0.0260	
≥35	3.19 (0.75, 13.51) 0.1150	
Race		0.4599
Mexican American	5.25 (1.03, 26.89) 0.0466	
Other Hispanic	1.41 (0.17, 11.62) 0.7522	
Non-Hispanic White	2.47 (0.64, 9.50) 0.1869	
Non-Hispanic Black	3.50 (0.71, 17.26) 0.1246	
Other Race	12.29 (1.58, 95.41) 0.0164	
Education level		0.3809
< High School	5.97 (1.16, 30.74) 0.0327	
≥ High School	3.14 (1.08, 9.15) 0.0360	
PIR		0.1972
<1.3	2.11 (0.62, 7.17) 0.2296	
≥1.3, <3.5	8.26 (1.77, 38.48) 0.0072	
≥3.5	3.48 (0.92, 13.11) 0.0657	
BMI (kg/m^2^)		0.1123
<25	1.47 (0.31, 6.83) 0.6264	
≥25	4.78 (1.55, 14.77) 0.0066	
Hypertension		0.4052
Yes	1.95 (0.36, 10.56) 0.4394	
No	3.69 (1.26, 10.81) 0.0170	
Hypercholesterolemia		0.2228
Yes	11.01 (1.13, 106.88) 0.0386	
No	3.40 (1.18, 9.84) 0.0238	
Gestational age		0.8991
1^st^ Trimester	2.58 (0.01, 772.90) 0.7444	
2^nd^ Trimester	25.70 (1.58, 418.98) 0.0226	
3^rd^ Trimester	29.21 (0.57, 1491.29) 0.0926	
Unclear	31.69 (1.59, 632.16) 0.0236	

### Diagnostic efficacy of TyG index for GDM

**Figure 2:**
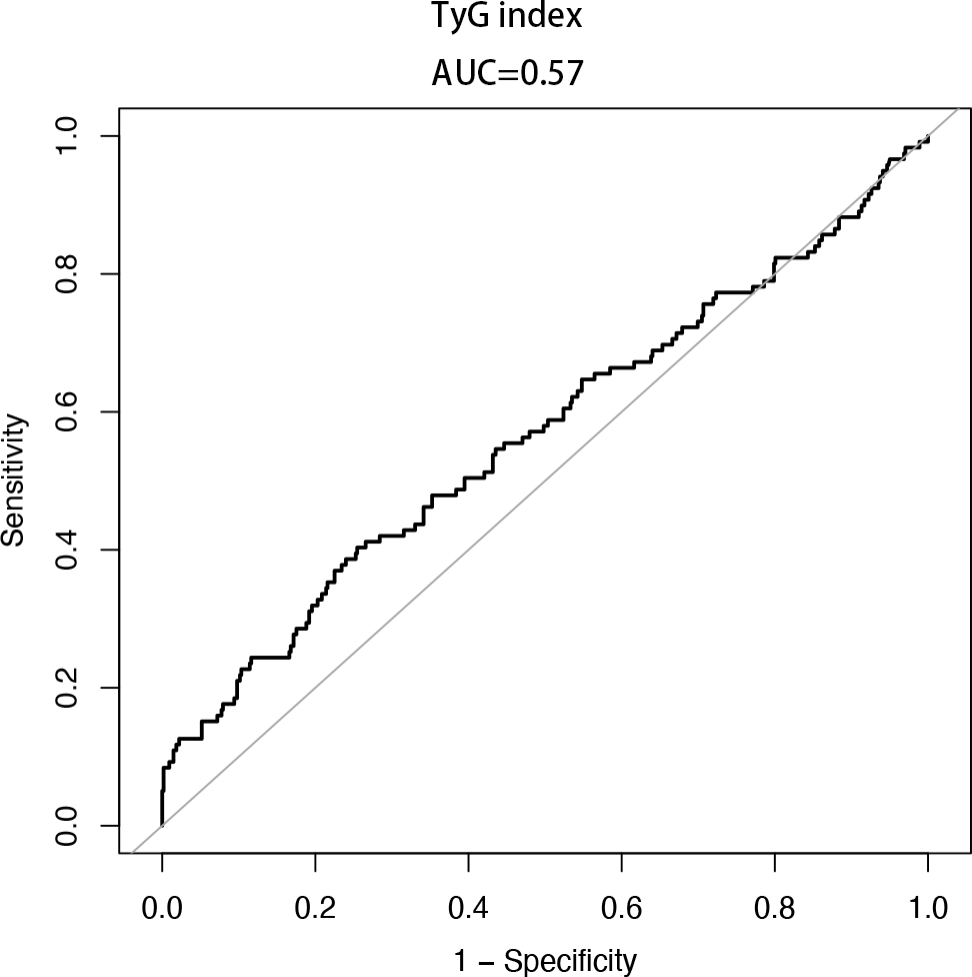
The ROC curve of the TyG index for diagnosing GDM. AUC = 0.57 (95% CI: 0.50-0.62, cut-off value is 9.07, sensitivity = 40.34%, specificity = 74.54%).

Analyzing the diagnostic effectiveness of the TyG index using a receiver operating characteristic (ROC) curve (**Figure 2**). The TyG index cut-off value for diagnosing GDM is 9.07 (AUC=0.57, 95% CI: 0.50-0.62, sensitivity=40.34%, specificity=74.54%). AUC values higher than 0.5 are regarded as having diagnostic utility.

## Discussion

This study further explored the correlation between TyG index and GDM using the nationally representative NHANES database. According to the results, pregnant women in the highest quartile of the TyG index had a 2.92-fold increased risk of developing GDM compared to those in the lowest quartile. The association between gestational diabetes mellitus and TyG index remains significant even after adjusting for all possible confounders, while the AUC of TyG index for predicting GDM was 0.57. It is noteworthy that the present study is the first to report a linear positive association between TyG index and GDM after adjusting for confounding variables, utilizing a population of pregnant women from the NHANES database from 1999 to 2020. Furthermore, the results of the subgroup analyses demonstrate the robustness of our findings.

The TyG index is a composite biochemical indicator that reflects the integrated influence of blood lipids and glucose. It has been reported to demonstrate high sensitivity in identifying insulin resistance among healthy individuals (11). Li et al. (18) conducted a cohort study that demonstrated a distinct association of the TyG index with an elevated probability of incident diabetes, suggesting its potential as a predictive indicator for type 2 diabetes mellitus (T2DM). The TyG index has been shown to be valuable in various cardiovascular disease types, including stable coronary artery disease, acute coronary syndromes, in-stent restenosis, arterial stiffness, coronary artery calcification, and heart failure (19). Meanwhile, multiple research investigations have revealed a strong correlation between the various factors of the TyG index and the risk of cardiovascular disease occurrence in both normal-weight individuals (20) and those suffering from non-alcoholic fatty liver disease (21). In a study of pregnant women, Pan et al. (22) observed that the TyG index in the early trimester of pregnancy is closely associated with the development of gestational hypertension and adverse pregnancy outcomes. Moreover, recent cohort studies have demonstrated a statistically significant association between the TyG index and the risk of developing gestational diabetes mellitus (GDM), indicating its efficacy as a predictor for both GDM and large for gestational age newborns (12, 23, 24).

GDM’s pathophysiology involves beta cell dysfunction and tissue insulin resistance during pregnancy. Elevated glucose levels can trigger islet beta cells to create reactive oxygen species, leading to oxidative stress and cell dysfunction, culminating in insulin resistance (25). Pregnant women, especially those with GDM, often exhibit elevated serum triglyceride levels, potentially impairing pancreatic beta cell function (26, 27). Early identification of GDM risk using reliable insulin resistance indicators is crucial to prevent adverse consequences. The TyG index is considered a cost-effective and efficient indicator of insulin resistance compared to other methods like Homeostatic Model Assessment for Insulin Resistance (HOMA-IR), as it eliminates the need for measuring insulin levels (28, 29). Its advantage lies in its ability to be derived from a single sample, making it highly suitable for various clinical applications (30).

Age, race, obesity, inheritance, smoking, and various risk factors collectively contribute to insulin resistance or GDM occurrence (31, 32). In this investigation, we comprehensively considered age, race, BMI, history of hypertension, hypercholesterolemia, alcohol use, smoking status, and gestational age as stratifying variables. Subgroup analysis and interaction tests consistently demonstrated correlations across different groups. Interestingly, our results from the subgroup analysis revealed that individuals aged <35 years, Mexican Americans, and those with a BMI ≥25 kg/m² exhibited a higher risk of GDM. BMI and age, two important characteristics related with insulin resistance, have a major influence on the development of GDM. It has been observed that maternal pre-pregnancy obesity substantially increases the risk of developing GDM, with an odds ratio (OR) of 1.63 (95% CI: 1.320-2.019), whereas the adjusted OR for older mothers (≥35 years) was 1.45 (95% CI: 1.184-1.776). This suggests that while age is indeed a risk factor for GDM, BMI exerts a greater influence on its development (33). Moreover, BMI serves as a crucial indicator for GDM in pregnant women with polycystic ovary syndrome (PCOS). Previous reports have highlighted that pregnant women affected by PCOS face an elevated risk of developing GDM if their BMI exceeds 28 kg/m² (34). However, the results of our analysis showed that a higher maternal TyG index during the first trimester of pregnancy did not significantly associate with GDM, raising concerns about using the TyG index as an early predictor of GDM. This finding aligns with a recent meta-analysis, suggesting that triglyceride variations during pregnancy and individual differences may contribute to these results (35). The limitations of the TyG index, derived from static measurements of fasting glucose and triglycerides, might not fully capture the intricate interactions of insulin sensitivity, insulin secretion, and glucose metabolism as assessed through oral or intravenous glucose tolerance tests (22, 36). Moreover, various factors affecting triglyceride metabolism, such as nutrition, alcohol use, antioxidants, medications, liver function, and genetic variations, could also impact the TyG index (19, 37, 38).

The predictive ability of the TyG index for GDM has been extensively investigated by multiple researchers. Liu et al. (24) reported an AUC of 0.686 (95% CI: 0.615-0.756) for the TyG index in predicting the risk of GDM. Similarly, Li et al. (12) demonstrated the potential of the TyG index in detecting the risk of GDM, with an AUC of 0.637 (95% CI: 0.626~0.649). Another study by Khan et al. (39) found that the TyG index exhibited the highest AUC, surpassing HbA1c and other biomarkers, with a predictive AUC of 0.712 (95% CI: 0.631-0.793) for GDM. In our study, we observed a limited effectiveness of the TyG index in predicting GDM, with an AUC of 0.57 (95% CI: 0.50-0.62). Differences in the reported ROC curves for the diagnosis of GDM using the TyG index may arise from various factors, including disparities in study populations, sample sizes, calculation methods, and reference standards utilized to diagnose GDM. Notably, in our study, the use of fasting blood glucose rather than the oral glucose tolerance test (OGTT) as the reference standard for GDM may have contributed to the observed discrepancy. While the TyG index as a composite indicator seems to reflect insulin resistance in pregnant women, further research is indispensable to fully explore its potential for early prediction of gestational diabetes.

Acknowledging the limitations of our research is essential. Firstly, the cross-sectional design using data from the NHANES database prevented us from establishing a direct causal association between the TyG index and the risk of developing GDM during pregnancy. Secondly, GDM recognition in this study relied solely on fasting plasma glucose, rather than using the more comprehensive OGTT. Several studies have indicated that our GDM categorization based only on fasting glucose may have an approximate 26% misclassification rate (40), potentially leading to an underestimation of GDM instances and influencing result interpretation. Thirdly, due to the cross-sectional design of the NHANES, all variable measurements were taken at a single point during a woman’s pregnancy, and trimester verification relied on pregnant women’s self-reports. Additionally, to avoid biased results, we did not compare the TyG index and HOMA-IR as distinct risk parameters for GDM, as the data for insulin levels in pregnant women had a substantial number of missing values. Future research is necessary to better predict GDM and its complications by incorporating the TyG index along with other clinical and biochemical parameters. Investigating potential mechanisms underlying the association between the TyG index and GDM, including the roles of adipokines, oxidative stress, inflammation, and insulin resistance, is also crucial. Despite these limitations, our study demonstrates a correlation between the gestational TyG index and GDM.

## Conclusion

The present research demonstrated a positive correlation between the TyG index and gestational diabetes in pregnant women in the US. However, the diagnostic validity of TyG index for GDM is limited. Further investigation is needed to fully explore the potential of the TyG index as a predictor of GDM risk in early pregnancy.

## Data availability statement

Publicly available datasets were analyzed in this study. This data can be found here: https://wwwn.cdc.gov/nchs/nhanes.

## Author contributions

YZ: conceptualization, statistical analysis, manuscript writing and editing. LY: methodology, reviewing and editing. XY: statistical analysis, reviewing. DZ: conceptualization, methodology, reviewing and editing. All authors contributed to the article and approved the submitted version.
